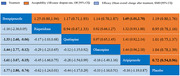# Efficacy and acceptability of atypical antipsychotics for behavioral and psychological symptoms of dementia: a systematic review and network meta‐analysis

**DOI:** 10.1002/alz.083587

**Published:** 2025-01-03

**Authors:** Wenqi Lyu, Weihong Kuang

**Affiliations:** ^1^ Mental Health Center, West China Hospital, Sichuan Univerisity, Chengdu China; ^2^ Mental Health Center, West China Hospital, Sichuan University, Chengdu China

## Abstract

**Background:**

Behavioral and psychological symptoms of dementia (BPSD) are highly prevalent in people living with dementia. Atypical antipsychotics (AAPs) are commonly used to treat BPSD, but their comparative efficacy and acceptability are unknown.

**Methods:**

This study was conducted following the guidelines of the Preferred Reporting Items for Systematic Review and Meta‐Analysis (PRISMA). Stata/SE (version 15.1) in a frequentist framework was used for the data analysis. The standard mean difference (SMD) was used to pool the fixed effect of continuous outcomes. We calculated odds ratios (ORs) with corresponding 95% credible intervals (CI) for the categorical variable. Relative treatment rankings were reported with mean ranks and the surface under the cumulative ranking curve.

**Results:**

Twenty RCTs with a total of 6374 individuals containing 5 types of AAPs (quetiapine, olanzapine, risperidone, brexpiprazole, and aripiprazole) with intervention lengths ranging from 6 weeks to 36 weeks were included in this NMA. For the efficacy outcome, compared with the placebo, brexpiprazole (SMD = ‐1.77, 95% CI ‐2.80 to ‐0.74) was more efficacious, and brexpiprazole was better than quetiapine, olanzapine, and aripiprazole. Regarding acceptability, only aripiprazole (OR = 0.72, 95% CI 0.54 to 0.96) was better than the placebo, and the brexpiprazole (OR = 1.65, 95% CI 1.01 to 2.70) was worse than aripiprazole. In terms of tolerability, olanzapine was worse than placebo (OR = 6.02, 95% CI 2.87 to 12.66), risperidone (OR = 3.67, 95% CI 1.66 to 8.11), and quetiapine (OR = 3.71, 95% CI 1.46 to 9.42), while aripiprazole was better than olanzapine (OR = 0.25, 95% CI 0.08 to 0.78).

**Conclusion:**

Brexpiprazole has shown great potential efficacy in the treatment of BPSD, with the highest acceptability of aripiprazole and the worst tolerability of olanzapine. The results of this study may be used to guide decision‐making.